# New developments in the pathology of malignant lymphoma. A review of the literature published from September–August 2017

**DOI:** 10.1007/s12308-017-0310-2

**Published:** 2017-11-29

**Authors:** J. H. van Krieken

**Affiliations:** Department of Pathology, Radboud University Medical Centre, P.O. Box 9101, 6500 HB Nijmegen, Netherlands

## Introduction

As I wrote in the editorial in this issue [1], it is rare to see single-authored original articles in the medical literature, but in this review, there is an exception. I.J. Miller retrieved 46 cases of Epstein Bar virus (EBV) positive lymphomas from the archives of his laboratory and looked carefully at the distribution of EBV early RNA (EBER)-positive cells [2]. I imagine that he or she was reviewing a case and wondered what it means that the number of EBER-positive neoplastic cells is so variable. It is a question that was commonly asked to me at “meet the professor” sessions or by residents and my answer was often that it might be technical, but that I really do not know. I never took the step to look into this issue a bit deeper and am happy that Miller did. Of the 46 cases, 7 had non-uniform staining among the neoplastic cells. Four of those cases showed a uniform admixture of EBER+ and EBER− cells, compatible with the prevailing theory of episomal EBV loss with cell replication. However, three cases showed a partial and zonal pattern which suggests that EBV infection occurred after the lymphoma was already established. In case 1, an EBV-follicular lymphoma (FL) and an EBV+ diffuse large B cell lymphoma (DLBCL) of activated B cell type (ABC) were contiguous in a lymph node. Both components showed a BCL2 translocation by fluorescence in situ hybridization (FISH). In case 2, a DLBCL of germinal center type (GCB) in an human immunodeficiency virus (HIV) positive patient contained clusters of EBER+ lymphoma cells with the morphology of Reed-Sternberg (RS) cells. In case 3, an ulcerated and perforated DLBCL in the stomach there was a superficial swath of EBER+ neoplastic cells accompanied by a relative absence of reactive T cells. In all 3 cases, the cells in EBER+ areas expressed latent membrane protein-1 and were strongly positive for CD30. Miller concluded that these three cases suggest that in a subset of EBV+ DLBCLs, EBV infection may not be the “first hit.” A nice piece of work, which indeed can be done by a single person. Nevertheless, it is my experience that discussion on data improve the quality of the research, so I welcome multi-authored articles!

## Biology of lymphoma

### Hodgkin lymphoma

When there are new scientific breakthroughs in the biology of cancer, classical Hodgkin’s lymphoma (cHL) is often early in the line of tumor types that are tested for the new insights. It was therefore to be expected that the possibility to investigate the topographical organization in tumors [3] is also applied in HL, because signaling between programmed cell death protein 1 (PD-1) and the PD-1 ligands (PD-L1, PD-L2) is essential for RS-cells to evade anti-tumor immunity in cHL. PD-L1 is also expressed by non-malignant tumor-associated macrophages (TAMs). Carey et al. [4] used multiplex immunofluorescence and digital image analysis to examine the topography of PD-L1+ and PD-1+ cells in the tumor microenvironment (TME) of cHL and demonstrate that the majority of PD-L1 in the TME is expressed by the abundant PD-L1+ TAMs which physically co-localize with PD-L1+ HRS cells in a microenvironmental niche. PD-L1+ TAMs are enriched for contacts with T cells and PD-L1+ HRS cells are enriched for contacts with CD4+ T cells, a subset of which are PD-1+. These data define a unique topology of cHL in which PD-L1+ TAMs surround HRS cells and implicate CD4+ T cells as a target of PD-1 blockade. This work shows the importance of studying neoplastic cells in their TME and not only as cell lines or by flow-cytometry. Koh et al. [5] also came to that understanding and, based on recent studies that reported the associations between PD-L1 or PD-L2/PD-1 pathways and pro-angiogenic genes, examined the relationship of PD-L1, PD-L2, PD-1, VEGF expression, and microvessel density (MVD) in cHL. Diagnostic tissues from 109 patients were evaluated by for PD-L1, PD-L2, PD-1, VEGF expression, and for CD31 expression as a measure of MVD. There was a positive correlation between PD-L1 and VEGF expression and additionally between PD-L2 and VEGF expression. The mean MVD in tumors positive for both PD-L1 and VEGF was higher than the mean MVD in tumors negative for both markers. The high PD-1 expression group had a lower 5-year overall survival rate than the low PD-1 expression group. These data confirm the positive correlations between PD-L1, VEGF, and MVD. Actually, this work would have gained strength when the method used by Carey would have been used. The same holds true for Vrzalikova et al. [6] who show that a subset of HL displays altered expression of sphingosine-1-phosphate (S1P) receptors (S1PR)s. S1P activates phosphatidylinositide 3-kinase (PI3-K) in these cells, mediated by the increased expression of S1PR1 and the decreased expression of S1PR2. They also showed that genes regulated by the PI3-K signaling pathway in HL cell lines significantly overlap with the transcriptional program of primary HRS cells. Genes upregulated by the PI3-K pathway included the basic leucine zipper transcription factor, ATF-like 3 (BATF3), which is normally associated with the development of dendritic cells. Immunohistochemistry (IHC) confirmed that BATF3 was expressed in HRS cells of most HL cases. In contrast, in normal lymphoid tissues, BATF3 expression was confined to a small fraction of CD30-positive immunoblasts. Knockdown of BATF3 in HL cell lines revealed that BATF3 contributed to the transcriptional program of primary HRS cells, including the upregulation of S1PR1. These data suggest that disruption of this potentially oncogenic feedforward S1P signaling loop could provide novel therapeutic opportunities for patients with HL.

Even deeper insight in the TME and the consequences of it, is given by Cohen et al. [7]. EBV is present in neoplastic cells of 15% of Asian and Latin-American DLBCL patients. Eventhough a tolerogenic microenvironment was recently described in DLBCL, little is known concerning immunomodulatory features induced by EBV. It has been suggested that in HL EBV-specific cytotoxic T cells are increased but show immune exhaustion features. Hence, host immunity suppression may play a critical role in tumor progression. Cytokine and chemokine transcripts expression and immunophenotype analysis showed that EBV infection was associated with increased gene expression of immunosuppressive cytokine (IL-10) together with increased CD8+ T cells and granzyme B+ cytotoxic effector cells. However, in EBV+ and EBV-DLBCL cases, the PD-1 expression suggests that this specific response is present in a tolerogenic milieu. High PD-1+ cell counts, EBV presence, and low CCL22 expression were associated with worse survival, supporting the hypothesis that a EBV-specific response is mounted locally and its inhibition by, for example PD-1+ cells, may negatively affect outcome.

### B cell lymphomas

In this series of reviews, I have separate headings for HL and B cell lymphoma (BCL), although HL actually is a very special form of the latter. Adams et all [8] investigated in various forms of BCL the expression of BCLW, an anti-apoptotic BCL-2, recently showed by them to be overexpressed in DLBCL and Burkitt lymphoma (BL). They performed a large-scale gene expression analysis of datasets comprising approximately 2300 lymphoma patient samples and validated the data by qRT-PCR and IHC. They report that BCLW is significantly overexpressed in aggressive and indolent lymphomas, including DLBCL, BL, FL, mantle cell lymphoma (MCL), marginal zone lymphoma (MZL), and HL. BCLW expression was negatively correlated with that of BCL2 in most lymphoma types, but in FL, BCLW was overexpressed as frequently as BCL2. The authors suggest that based on their data BCLW may be equally as important in lymphomagenesis as BCL2. However, because BCL-2 overexpression is related to a translocation in FL, it seems to me that that is actually more important in oncogenesis.

Jiang et al. [9] investigated the function of inhibin βA (INHBA) in DLBCL. They show that both mRNA and protein levels of INHBA are downregulated in primary DLBCL tissues, irrespective of GCB or ABC subtype, compared to those in benign tonsils. The low level of INHBA in patients with de novo DLBCL was correlated with reduced overall and progression-free survival. Ectopic expression of INHBA in DLBCL cell lines (OCI-Ly01 and SUDHL-10) resulted in reduced cell proliferation, increased spontaneous apoptosis and, an arrested cell cycle and suppressed xenograft tumor growth. Moreover, INHBA enhanced the chemosensitivity of DLBCL cells. Although the mechanism behind this altered expression remains to be clarified, these results provide an indication that INHBA functions as a tumor suppressor in DLBCL and may therefore be a target for treatment. Next to this classic approach to unravel mechanisms important in oncogenesis, a whole new approach has recently become in vogue, made possible due to developments in high-throughput genetics and novel bioinformatic approaches. Karube et al. [10] have integrated the results of targeted next-generation sequencing of 106 genes and genomic copy number alterations (CNA) in 150 DLBCL patients and correlated the results to clinical data. The significant findings were validated in an independent cohort of 111 patients. GCB and ABC subtypes DLBCL had a differential profile of mutations, altered pathogenic pathways and CNA. Mutations in genes of the NOTCH pathway and tumor suppressor genes (TP53/CDKN2A), but not individual genes, indicated an unfavorable prognosis, confirmed in an independent validation cohort. Gene expression profiling showed that tumors with NOTCH pathway mutations had a significant modulation of downstream target genes, emphasizing the relevance of this pathway in DLBCL. An in-silico drug discovery analysis recognized 69 (46%) cases carrying at least one genomic alteration considered a potential target of drug response according to early clinical trials or preclinical assays in DLBCL or other lymphomas. The authors conclude that these data identify relevant pathways and mutated genes in DLBCL and discovers potential targets for new intervention strategies. However, in my opinion it remains relevant to confirm in-silico data with preclinical in vitro evidence before patients can be treated.

A similar approach was used by Bouska et al. [11]. They performed high-resolution structural and functional genomic analysis of adult BL and high-grade B cell lymphoma with a BL gene signature (adult-molecularly defined BL [mBL]) and show that the MYC-ARF-p53 axis is the primary deregulated pathway. Adult-mBL had either unique or more frequent genomic aberrations (del13q14, del17p, gain8q24, and gain18q21) compared with pediatric-mBL, but shared commonly mutated genes. Mutations in genes promoting the B cell receptor (BCR) → PI3K pathway (*TCF3* and *ID3*) did not differ by age of the patient, whereas effectors of chronic BCR → NF-κB signaling were associated with adult-mBL. A subset of adult-mBL had a *BCL2* translocation and mutation and elevated *BCL2* mRNA and protein expression, but had a mutation profile similar to mBL. These double-hit lymphomas may have arisen from a tumor precursor that acquired both *BCL2* and *MYC* translocations and/or *KMT2D* (*MLL2*) mutation. Gain/amplification of *MIR17HG* and its paralogue loci was observed in 50% of adult-mBL. They went on to perform in vitro confirmation and suggest a *miR-17* ∼ *92*’s role in constitutive activation of BCR signaling and sensitivity to ibrutinib. Overall integrative analysis identified an interrelated gene network affected by CNA and mutations, leading to disruption of the p53 pathway and the BCR → PI3K or NF-κB activation, which can be further explored in vivo by small-molecule inhibitors for effective therapy in adult-mBL.

With all these new approaches that might lead to effective precision drugs, we should keep in mind that presently most cures for cancers still come from surgeons and classic chemotherapy. Wei et al. [12] look at chemicals that have a relation which effective chemotherapy. They show that genomes of BCL contain high levels of abasic sites presumably created during the repair of uracils through base-excision repair. Furthermore, three alkoxyamines with an alkyne functional group link covalently to abasic sites in DNA and kill immortalized cell lines created from BCL, but not other cancers. They also do not kill normal B cells. Treatment of cancer cells with one of these chemicals causes strand breaks, and the sensitivity of the cells to this chemical depends on the ability of the cells to go through the S phase. Other alkoxyamines that also link to abasic sites—but lack the alkyne functionality—do not kill cells from BCL. This shows that the ability of alkoxyamines to covalently link to abasic sites is insufficient for their cytotoxicity and that the alkyne functionality may play a role in it. The authors conclude that these chemicals violate the commonly accepted bio-orthogonality of alkynes and are attractive prototypes for anti-B cell cancer agents. This new approach to “old” drugs might result in more precision for even classic chemotherapy.

### T cell lymphoma

Somatic G17V RHOA mutations are present in 50–70% of angioimmunoblastic T cell lymphoma (AITL). The mutant RHOA lacks GTP binding capacity, suggesting defects in the classical RHOA signaling. Fujisawa et al. [13] discovered a novel function of the G17V RHOA: VAV1 was identified as a G17V RHOA-specific binding partner via high-throughput screening. Furthermore, binding of G17V RHOA to VAV1 augmented its adaptor function through phosphorylation of 174Tyr, resulting in acceleration of T cell receptor (TCR) signaling. Enrichment of cytokine and chemokine-related pathways was also evident by the expression of G17V RHOA. They also identified VAV1 mutations and a new translocation, VAV1-STAP2, in seven of the 85 RHOA mutation-negative samples (8%), whereas none of the 41 RHOA mutation-positive samples exhibited VAV1 mutations. In vitro, dasatinib, a multikinase inhibitor, efficiently blocked the accelerated VAV1 phosphorylation and the associating TCR signaling by both G17V RHOA and VAV1-STAP2 expression. Phospho-VAV1 staining was demonstrated in the clinical specimens harboring G17V RHOA and VAV1 mutations at a higher frequency than those without. These findings indicate that the G17V RHOA-VAV1 axis may provide a new therapeutic target in AITL.

## Epidemiology of lymphoma

The only way to get an idea on the clinical features of rare lymphomas are retrospective analyses of large patient collections. In this review, a few nice examples.

Tokuhira et al. [14] collected the data from 62 patients with methotrexate-induced lymphoproliferative disorders (MTX-LPDs). Forty-three patients showed regression but 14 of them (33%) relapsed (median disease free survival 11 months). Surprisingly, there was no difference in overall survival (OS) between the patients with and without regression, but relapse-free patients had a superior OS. The presence of EBV was associated with longer MTX treatment and was also related to clinical manifestations.

Alonso-Álvarez et al. [15] used data from 1734 patients (800 males/934 females; median age 59 years), diagnosed with FL grades 1-3A, to investigate whether clinical features have changed in this rituximab era. With a median follow-up of 6 years, 106 patients developed a histologically confirmed transformation with a median time to transformation of 2.5 years. High-risk FL International Prognostic Index (IPI) and non-response to first-line therapy were associated with transformation. Seventy out of the 106 patients died of disease. Response to first-line therapy after transformation, autologous stem cell transplantation, and revised IPI were associated with survival. These data indicate a better outcome for FL patients after the introduction of rituximab. It is not clear whether primary splenic cases were included, which might be relevant given the data from Shimono et al. [16]. They investigated the clinicopathological features of 17 patients with primary splenic FL, as compared to data from 153 nodal FL cases. Hepatitis C virus (HCV)-positive status was significantly more common in patients with splenic FL than in the control patients. Ann Arbor stage III or IV and high-risk FLIPI were significantly less common in patients with splenic FL than in the control patients, but it must be noted that there is a definition bias here: by definition disseminated cases were excluded for the splenic group! Nevertheless, the overall and progression-free survival curves were not different between the groups. Actually, among the 17 patients with splenic FL, the progression-free survival was significantly worse in patients who underwent splenectomy without receiving postoperative chemotherapy than in those who did receive it. According to the authors, their results suggest that primary splenic FL should be considered different from systemic FL and that its management should also be different. However, in my opinion this is not really what their data show.

Paul et al. [17] were interested in outcome data of elderly patients with DLBCL, who are often underrepresented in published literature, while the incidence increases with age. They analyzed data of 1534 patients encompassing all adult age groups, enriched for the age ≥ 75 years. Transformed FL cases were included. Gender, centroblastic morphology, transformed FL, CD10 expression, and ABC subtype were significantly associated with age after correction for multiple testing and after adjusting for cohorts. Analysis of a subgroup points towards an association of MYC expression with age. These data indicate that biological features of DLBCL and transformed FL are associated with increasing age.

Virmani et al. [18] collected data from 74 patients with mycosis fungoides (MF) who are younger than 30 years old. Median age at diagnosis was 25 years, 65 of them (88%) presented with early stage disease and/or variants of MF (*n* = 44 [59%]), leading to a median delay in diagnosis of 2.5 years. Hypopigmented MF (*n* = 27 [37%]) was the most common variant, affecting predominantly African American (44 vs 19%) and younger (20 vs 26 years) patients. All patients with hypopigmented MF presented with early stage disease and were less likely to develop progressive disease (PD) compared to patients with other variants (11 vs 34%). Nineteen patients (26%) developed PD during a median follow-up of 3.5 years, which was associated with advanced-stage disease (89 vs 17%), age higher than 20 years (31 vs 13%), African American race (53 vs 20%), and poikilodermatous presentation. OS was good (97% at 5years, 96% at 10 years) despite the delay in diagnosis and atypical presentation.

Suzuki et al. [19] investigated the clinicopathological features of 46 patients with CD4^+^ and/or CD56^+^ immature hematolymphoid malignancy (iHLM), including blastic plasmacytoid dendritic cell neoplasm (BPDCN). They categorized these cases into three groups and performed IHC with three plasmacytoid dendritic cell (pDC) markers [CD123, CD303, and T cell leukemia/lymphoma (TCL1)]. The three groups included cutaneous BPDCN (*n* = 35), non-cutaneous BPDCN (*n* = 6), and non-BPDCN-type CD56^+^ neoplasms (*n* = 5). Compared to non-cutaneous BPDCN, cutaneous BPDCN was associated with an older median age at onset (72 vs 45 years) and positivity for CD4, CD123, and 2-3 pDC markers (89 vs 50%). Cutaneous BPDCN was divided into terminal deoxynucleotidyl transferase (TdT) positive and TdT negative subgroups, which did not differ in prognosis, although TdT-positive cases showed a lower median onset age (66 vs 79 years) and higher frequency of extracutaneous lesions. Compared to the BPDCN groups, non-BPDCN-type CD56^+^ neoplasm cases showed higher cytoplasmic CD3 positivity and less frequent BCL-2 expression, and absence of cutaneous lesions. There were no survival differences. The authors conclude that their data support the recognition of cutaneous BPDCN as a homogenous entity, in contrast to the non-cutaneous form. However, the number of patients was too small to characterize non-BPDCN-type CD56^+^ neoplasms.

## Defining entities

### Hodgkin lymphoma

The difference between cHL and nodular lymphocyte predominant HL is important, but not always easy. Kezlarian et al. [20] investigated whether staining for GATA3, a T cell transcription factor involved in T cell maturation overexpressed in cHL cells, might be helpful. GATA3 was positive in 80% of cHLs (*n* = 49) but the extend of positivity and intensity of staining varied greatly. GATA3 was negative in all NLPHLs [17], EBV+ DLBCL (*n* = 2), T cell rich BCL (*n* = 2), and DLBCL (*n* = 72). Interestingly, the single gray zone lymphoma and 3/4 primary mediastinal DLBCL (PMBCL) were positive, a finding that further highlights similarities between cHL and PMBCL. The authors conclude that nuclear expression of GATA3 can be used to delineate cHL from NLPHL because GATA3 positivity excludes NLPHL with 100% negative predictive value. However, as c0% of CHL can be negative for GATA3, cHL cannot be ruled out with negative GATA3.

Another issue in NLPHL are aberrant findings. Untanu et al. ([21]) used data from a clinical trial and included 168 cases of localized NLPHL. Fifty-eight (35%) cases showed only typical nodular growth pattern. The remainder showed mixtures of histologies, including extranodular large B cells, T cell-rich nodular pattern (*n* = 55; 33%) with diffuse T cell-rich pattern, and diffuse B cell pattern. These had a somewhat lower event free survival but no decreased OS. Cytoplasmic IgD was found in 65 of 130 tested (50%), but was not associated with survival either. Seventeen (10%) expressed CD30, with no adverse effect. These data show that variant histology and phenotype is common in pediatric NLPHL, but does not affect overall survival.

### B cell lymphomas

It is well known that some gastric extranodal (e) MZL do not react to *Helicobacter pylori* (*H. pylori*) eradication and that t(11;18) plays a role. Iwamuro et al. [22] reviewed FISH data from146 patients with eMZL. Complete response was obtained in 61/88 patients without t(11;18) and two copies of MALT1 (69%), 22/27 in patients with t(11;18) (82%), and 21/31 in patients with extra copies of MALT1 (68%). Of note, generally, patients without translocation were treated with *H. pylori* eradication alone and patients with translocation with radiotherapy alone.

Zheng et al. [23] further characterized the presence of primary central nervous system DLBCL (PCNS-DLBCL) mutations in MYD88 and CD79B as well as DNA methyltransferase (MGMT) methylation. MYD88 mutations were identified in 69% (35 of 51 cases), with L265P being the most frequent mutation. Mutations other than L265P were identified in 22% of cases, of which eight were novel MYD88 mutations. Of the mutated cases, 18% had a homozygous/hemizygous MYD88 mutation, which has not been previously reported in PCNS-DLBCL. CD79B mutations were found in 6 of 19 cases (32%), all in the Y196 mutation hotspot. MGMT methylation was observed in 37% (20 of 54 cases). There was no significant difference in median OS between the wild type and mutated MYD88 cases, or between methylated and unmethylated MGMT cases. However, a significant difference was noted in median OS between the wild type and mutated CD79B cases. Nevertheless, the detection of mutations in MYD88 might be relevant. Nielsen et al. [24] investigated whether patients with mutated MYD88 lymphomas have an immunological response to the abnormal protein. They assessed T cells from 19 healthy donors for recognition of three common driver mutations in lymphoma: *MYD88* ^*L265P*^, *EZH2* ^*Y641F*^, and *EZH2* ^*Y641N*^. Donors collectively expressed the ten most prevalent HLA class I alleles, including HLA-A*02:01. Peripheral blood T cells were primed with peptide-loaded dendritic cells (DC), and reactive T cells were assessed for recognition of naturally processed mutant versus wild type full-length proteins. They identified CD4 positive T cells against EFISE**N**CGEII from EZH2^Y641N^ (presented by HLA-DRB1*13:02) and CD8^+^ T cells against R**P**IPIKYKA from MYD88^L265P^ (presented by HLA-B*07:02). They did not detect R**P**IPIKYKA-specific T cells in seven other HLA-B*07:02-positive donors, including two lymphoma patients. Thus, healthy donors harbor T cells specific for common driver mutations in lymphoma. However, such responses appear to be rare due to the combined limitations of antigen processing, HLA restriction, and T cell repertoire size, highlighting the need for highly individualized approaches for selecting targets. Whether these healthy donors produce these cells because they had mutated cells that have effectively been removed by the immune response remains an interesting possibility.

Primary bone (PB) DLBCL is rare and has a favorable prognosis, but the underlying biological mechanisms remain unknown. Li et al. [25] analyzed the clinicopathologic features of 160 patients with PB-DLBCL and compared their findings with those of 499 non-osseous DLBCL. PB-DLBCL patients were less frequently of elderly age, had less often B symptoms, elevated serum lactate dehydrogenase levels, and high IPI at diagnosis. The lymphoma was more frequently of the GC-subtype (approximately 90%). The 5-year progression-free and overall survival rates of PB-DLBCL patients were 80 and 93%, respectively, superior to both GCB and ABC subtypes of non-osseous DLBCL. PB-DLBCL had similar survival rates as the centrocyte-origin (CC) subtype of DLBCL-GCB classified by the B cell-associated gene signature algorithm. This was confirmed by analyzing a small subset of PB-DLBCL, which had a gene expression profiles resembling that of non-osseous DLBCL-GCB-CC, and distinct from other DLBCL cell-of-origin, especially the centroblast-origin (CB) subtype. These results demonstrate that PB-DLBCL is clinically distinct, and the cell-of-origin of PB-DLBCL stems from centrocytes in the GC that are biologically attributed for the favorable prognosis of PB-DLBCL. The cell of origin theory forms the basis of the classification of tumors and thus for the definition of a process knowledge of the cells from which it is derived is quite valuable. Pham-Ledard et al. [26] investigated the status of the immunoglobulin genes of primary cutaneous (pc) DLBCL, leg-type (lt) to see whether they could confirm that this process derives from germinal center-experienced B lymphocytes, as is suggested by the immunophenotype (BCL2+, MUM1+, BCL6 +/−). Using the BIOMED2 protocol with VH leader primers clonality testing with sequencing was done on frozen skin biopsies from 14 patients. The clonal DNA IGHV sequence of the tumor was aligned and compared with the closest germline sequence and homology percentage was calculated. A functional monoclonal sequence was observed in 14 cases as determined for IGHV [10], IGLV [2], or IGKV [3]. IGV mutation rates were high (> 5%) in all cases but one, with conservation of superantigen binding sites. Features of selection pressure were identified in 11/12 interpretable cases, more frequently negative (75%) than positive (25%). Intraclonal variation was detected in three of eight tumor specimens with a low rate of mutations. Surface immunoglobulin was IgM in 12/12 cases. FISH analysis of the IGHM locus, deleted during class switching, showed heterozygous IGHM gene deletion in half of the cases. The genomic PCR analysis confirmed the deletions within the switch μ region. IGV sequences were highly mutated but functional, with negative features of selection pressure suggesting one or more germinal center passage(s) with somatic hypermutation, but superantigen (SpA) binding sites conservation. Genetic features of class switch were observed, but on the non-functional allele and co-existing with primary isotype IgM expression. These data indicate that the cell-of origin is a germinal center-experienced and superantigen-driven selected B cell, in a stage between germinal center B cell and plasma cell.

### Cutaneous lymphoma

The distinction between reactive and neoplastic lymphoproliferations of the skin is at times quite difficult. Fernandez-Pol et al. [27] tried to find markers for the distinction between subcutaneous panniculitis-like T cell lymphoma (SPTCL) and lupus erythematosus panniculitis (LEP), lesions that have clinical and histological overlap. Using IHC for the MYC oncoprotein on 23 cases of SPTCL (1 CD8 negative) and 12 cases of LEP. In SPTCL cases, the percentage of cells that were c-Myc positive ranged from 0.8 to 16%, with a mean of 5.0% and a median of 4.4%. In contrast, in the LEP cases, the percentage of c-Myc-positive cells in the cases ranged from 0.34 to 3.7%, averaged 1.4% and the median was 0.8%. The difference between the means of these two diagnostic categories was statistically significant; however, there is substantial overlap. FISH on four cases of SPTCL with a relatively high number of MYC-positive cells did not reveal MYC rearrangement or amplification. In my opinion, the additional value of this test therefore is limited. The conclusion of the authors that their work suggests that MYC that may play a role in the pathogenesis of SPTCL is not substantiated by the data.

Above, I described the mechanism of RHOA in AILT, but Leclair Alirkilicarslan et al. [28] used the mutations that occur in this gene in AILT in a more practical manner. The analyzed skin biopsies of 41 AILT patients for the expression of follicular helper T cell (TFH) markers, EBV, and the presence of RHOA (p.G17V) and IDH2 (p.R172K/S) mutations using allele-specific polymerase chain reaction. They categorized the cases into four distinctive patterns: [1] low-density lymphocytic perivascular infiltrates (*n* = 11), [2] dense perivascular infiltrates with atypical cells and occasional inflammatory cells (*n* = 13), [3] diffuse infiltrates reminiscent of AILT (*n* = 4), or [4] other aspects (*n* = 13). They observed variable expression of TFH markers (CD10 [50%], BCLB6 [84%], PD1 [94%], CXCL13 [84%], and ICOS [97.5%]) and EBV positive cells (26%). A TFH phenotype was identified in 82 and 73%, respectively, of cases with the most challenging patterns 1 and 2. TFH markers and EBV can thus help for diagnosis and are detected in samples with low-density infiltrates. Furthermore, they found RHOA G17V and IDH2 R172K/S mutations in the skin in 14/18 (78%) and 3/16 (19%) cases, respectively. The RHOA G17V mutation was identified in a proportion of biopsies with patterns 1 and 2, which represent a diagnostic challenge. The RHOA G17V mutation was detected both in the skin and lymph node (LN) biopsies in 7/9 (64%) cases, and in only the skin or the LN of one sample each. The frequency of RHOA G17V mutation was similar to that reported in LNs. These data indicate that especially RHOA mutation testing might be a sensitive diagnostic marker in the skin biopsies of patients with AILT, especially in cases with low-density infiltrates.

## New entities/subtypes

What makes an unusual feature in a lymphoma type just something to be known or the indication that one is dealing with a variant or even a different entity? For instance, the question whether cases that have morphological and phenotypical features of FL but lack a bcl-2 break are real FL has been discussed for years: is this a variant or not? Is it indicating just a special feature or indeed, as van den Brand et al. [29–31] argue, most of them are actually a different entity, nodal marginal zone lymphoma (NMZL). Zamò et al. [32] created exonic single-nucleotide variant (SNV) profiles of 28 t(14;18)-positive and 13 t(14;18)-negative FL, followed by integration of copy number changes, copy-neutral LOH with published gene-expression data as well as the assessment of immunoglobulin N-glycosylation sites. They find that typical FL mutations also affected t(14;18)-negative FL. Curated gene set/pathway annotation of genes mutated in either t(14;18)-positive or t(14;18)-negative FL revealed a strong enrichment of same or similar gene sets but also a more prominent or exclusive enrichment of immune response and N-glycosylation signatures in t(14;18)-negative FL. Mutated genes showed a high BCL2 association in both subgroups. Among the genes mutated in t(14;18)-negative FL, 555 were affected by copy number alterations and/or copy-neutral LOH and 96 were differently expressed between t(14;18)-positive and t(14;18)-negative FL (*P* < 0.01). N-glycosylation sites were detected considerably less frequently in t(14;18)-negative FL. The authors conclude that these results suggest a diverse portfolio of genetic alterations that may induce or regulate BCL2 expression or promote pathogenesis of t(14;18)-negative FL as well as a less specific but increased crosstalk with the microenvironment that may compensate for the lack of N-glycosylation. It is obvious a real pity that a comparison with NMZL was not made. Maybe this is the result from work that is more inventory of nature rather that hypothesis driven.

Igawa et al. [33] describe unusual morphological feature in MCL. In three cases, they find features of plasma cell-type Castleman disease (CD). The three patients were all men, ranging from 51 to 74 years in age, and they all presented with systemic lymphadenopathy with anemia, hypoalbuminemia, elevated serum levels of C-reactive protein, and polyclonal hypergammaglobulinemia. Lymph node biopsy specimens of the three cases showed histological features of plasma cell-type CD, including atrophic germinal centers and interfollicular plasmacytosis, with no light chain restriction. However, flow cytometric analysis demonstrated an abnormal B cell population with CD5 expression, and further analysis using cyclin D1 immunostaining highlighted a neoplastic component that was restricted to the mantle zone. These neoplastic cells were immunohistochemically positive for CD20, CD5, and SOX11, and negative for CD3, CD10, and HHV8. The Ki67 index was low. The authors indicate that these patients have indeed MCL with unusual morphological features, but in fact, their data point more to CD with maybe MCL in situ or combined MCL and CD. However, one must be careful with diagnosing from just images and description.

It is well known that a subset of MCL patients have a good prognosis without treatment, as we described already in 1996 [34]. However, it is still not common to have a wait and see policy in MCL, even in elderly patients with low proliferation in the tumor cells. Abrisqueta et al. [35] analyzed their population based registry and found 440 patients with MCL diagnosed between 1998 and 2014. Of their patients, 365 (83%) received early treatment and 75 (17%) were observed for more than 3 months without treatment. In the observation group, 54 (72%) patients had a nodal presentation, 16 (21%) a non-nodal presentation, and 5 (7%) had only gastrointestinal involvement. Characteristics associated with deferred treatment included good performance status, no B symptoms, low LDH, non-bulky disease, non-blastoid morphology, and lower Ki67 values. The median time to treatment in the observation group was 35 months (range 5–79), and 60 (80%) patients were observed beyond 12 months. The median OS was significantly longer in the observation group than in the early treatment group (72 vs 53 months, respectively). These data indicate again that also after 20 years, our data are confirmed and that a subgroup of patients with MCL can be safely observed without treatment without negatively impacting their outcomes. Eskelund et al. [36] address a further question in MCL: do all patients benefit from intensified treatment? They explore the prognostic value of recurrent genetic aberrations in diagnostic bone marrow (BM) specimens from 183 younger patients with MCL from the Nordic MCL2 and MCL3 trials, which represent current standard-of-care regimens. In a univariate model, mutations of *TP53* (11%) and *NOTCH1* (4%), and deletions of *TP53* (16%) and *CDKN2A* (20%), were associated with inferior outcome (together with MIPI, MIPI-c, blastoid morphology, and Ki67 higher than 30%); however, in multivariate analyses, only *TP53* mutations retained prognostic impact for OS, whereas *TP53* mutations and MIPI-c high-risk had independent prognostic impact on time to relapse. *TP53*-mutated cases had a dismal outcome, with a median OS of 1.8 years, and 50% relapsed at 1.0 years, compared to a median OS of 13 years for *TP53*-unmutated cases. *TP53* mutations were associated with Ki67 higher than 30%, blastoid morphology, MIPI high-risk, and inferior responses to both induction- and high-dose chemotherapy. These data indicate that *TP53* mutations identify a distinct and highly aggressive form of MCL with poor or no response to regimens including cytarabine, rituximab, and autologous stem cell transplant (ASCT). The authors suggest that patients with MCL should be stratified according to *TP53* status, and that patients with *TP53* mutations should be considered for experimental frontline trials exploring novel agents. Zlamalikova et al. [37] add significant data to this work. They present the complex analysis of the TP53 aberrations in 57 cases of MCL and 131 cases of DLBCL. The TP53 status was determined by functional analyses in yeast (FASAY) followed by cDNA and gDNA sequencing. The level of the p53 protein was assessed by immunoblotting and loss of the TP53-specific locus 17p13.3 was detected by FISH. Altogether, they detected 13 TP53 mutations among MCL cases (23%) and 29 TP53 mutations in 26 from 131 DLBCL cases (20%). The ratio of missense TP53 mutations was 77% in MCL and 83% in DLBCL. The frequency of TP53 locus deletion was rather low in both diseases, reaching 9% in MCL and 15% in DLBCL. The presence of TP53 mutation was associated with shorter OS and progression-free survival (PFS) in MCL. Among DLBCL cases, the TP53 mutations shortened both OS and PFS of patients treated with R-CHOP (rituximab, cyclophosphamide, doxorubicin, vincristine, and prednisone) and decreased both OS and PFS of patients with secondary DLBCL disease. It would be interesting to see how much overlap there is in patients in both studies and what really confers the lack of treatment response.

The cause of cancer remains often enigmatic, but sometimes agents that play a role have been identified. For instance, there are hepatitis C (HCV) associated lymphomas and Visco et al. [38] describe the features of HCV-positive DLBCL. They compared 44 HCV-positive cases of de novo DLBCL with 132 HCV-negative patients, matched for age, lactate dehydrogenase level and IPI at presentation. Compared to the HCV-negative controls, patients with HCV-positive DLBCL had differential expression of genes that regulate innate immune response and modulate apoptotic pathways have higher proliferative index and lack BCL2 translocations. Most important remains the fact that treatment of HCV infection can lead to disappearance of the lymphoma.

Wu et al. [39] compare the clinicopathological features of 17 cases of extranodal NK/T cell lymphoma of nasal type (NKTL) with cutaneous involvement with 14 patients with stage I, the extranasal variant (ENKTL), including seven in the skin, five in the gastrointestinal tract, and two in the central nervous system. The seven primary cutaneous (PCNKTL) cases were characterized by an older onset age (median, 76 vs 53 years) and a more favorable clinical course compared with the 17 patients with stages II-IV ENKTL that showed cutaneous involvement. The skin lesions in the PCNKTL group were distributed in the face or neck (*n* = 4) and limbs (*n* = 3) but not the trunk, which was most frequently affected (60%) in ENKTL group. Furthermore, the stage I cutaneous disease showed a female predominance (male–female, 2:5 vs 7:0) and a significantly more favorable survival compared with the non-cutaneous stage I ENKTL. According to the authors, suggest these results that PCNKTL constitute a distinct subgroup in the nasal-type lymphoma spectrum. The survival data however are not a good argument, since the groups are selected based on stage, of course an important prognosticator!

## Pitfalls in lymphoma diagnosis

TdT is an important marker of precursor neoplasia, but is also present in precursor T and B cells. One has therefore to be careful with interpretation. Wen et al. [40] evaluated the incidence and distribution of immature B lymphocytes co-expressing TdT and PAX-5, in pediatric and adult liver biopsies, to determine whether a normal hepatic immature B cells can be detected. They selected 41 pediatric and adult liver biopsies with a significant portal and/or sinusoidal hematolymphoid infiltrate and performed IHC for TdT and PAX-5. TdT-positive cells were detected in 40% of pediatric liver biopsies with a significant hematolymphoid infiltrate (4/10), which included all biopsies from neonates and infants under 9 weeks of age. In adults, immature B cell infiltrates were less common (6%, 2/31). Dual immunostaining was performed on two cases of neonatal hepatitis, which documented B cell lineage in at least a subset of TdT-positive cells, and there was no co-labeling with CD3. Therefore, immature B cells can be present in liver biopsies in a variety of clinical settings, most commonly in children, and the presence of a few TdT-positive cells cannot be considered specific for involvement by B-ALL. Further workup for B-ALL can be warranted if there is more extensive multifocal portal and/or sinusoidal involvement by blasts with TdT labeling.

## Prognostic/predictive factors in lymphoma

Kiesewetter et al. [41] tried to predict the value of lenalidomide in MZL. Because high expression of cereblon (CRBN) and MUM1 have been associated with better response rates in multiple myeloma patients treated with lenalidomide, they investigated CRBN/MUM1 expression assessed by IHC in 46 eMZL (13/46 gastric; 33/46 extragastric) patients treated with lenalidomide-based therapy: 54% showed high expression (CRBN+, ≥ 50% positive cells). In contrast to other reports, there was no difference in response rate in CRBN+ or MUM1 + cases nor in relapse rate and PFS but all three patients progressing on lenalidomide were CRBN+ and both patients completely lacking CRBN expression responded to treatment; nevertheless reliable prediction cannot be given.

According to Bielska et al. [42], there is an association of IKZF1 variants with DLBCL outcome. IKZF1 encodes a transcription factor involved in B cell maturation and differentiation. They genotyped 218 DLBCL patients and 715 unrelated controls. No difference was observed in the genotype distribution of the IKZF1 rs4132601 polymorphism between DLBCL patients and controls, but the 2-year PFS rate of patients with the IKZF1 TT genotype was 54% compared to 69% in those with the IKZF1 G+ genotypes.

Lee et al. [43] found that MyD88 mutation status did not correlate with overall survival (OS) in 165 patients with DLBCL post-autologous stem cell transplantation nor progression-free survival (PFS). Patients with non-GCB subtype had significantly worse OS from initial diagnosis and after ASCT. Not surprisingly, high IPI score was predictive of poor pre- and post-transplant PFS and post-transplant OS.

Leivonen et al. [44] compared alternative splicing (AS) and differentially expressed genes and exons in association with survival after chemo-immunotherapy, and between GCB and activated ABC DLBCLs in 38 clinically high-risk patients. They conclude that their results indicate that AS events are able to discriminate GCB and ABC DLBCLs and have prognostic impact in DLBCL. Quite a strong conclusion for such low numbers of patients.

Kato et al. [45] investigate 88 peripheral T cell lymphomas, not otherwise specified (PTCL-NOS for expression of cell adhesion molecule 1 (CADM1). CADM1 was expressed in 14 of 88 (16%) PTCL-NOS cases, and its expression was associated with C-C chemokine receptor type 4 (CCR4) expression and nuclear atypia. CADM1-positive PTCL-NOS cases (10/74) had a poorer prognosis than CADM1-negative cases (64/74). Multivariate analysis confirmed that CADM1 expression was an independent prognostic factor in PTCL-NOS.

## References

[1] van Krieken JH (2017) When to be an author? J. Hematopathology. 10.1007/s12308-017-0311-1

[2] Vasaturo A, Di Blasio S, Verweij D, Blokx WA, van Krieken JH, de Vries IJ, Figdor CG (2017). Multispectral imaging for highly accurate analysis of tumour-infiltrating lymphocytes in primary melanoma. Histopathology.

[3] Miller IJ 2017 Epstein Barr virus infection can be a secondary event in B-cell lymphomas: a review of 338 cases and a novel finding of zonal EBER+ tumor cells showing features of progression from underlying EBV-negative lymphoma. Appl Immunohistochem Mol Morphol. 10.1097/PAI.0000000000000562, 1 10.1097/PAI.0000000000000562 28800008

[4] Carey CD, Gusenleitner D, Lipschitz M, Roemer MGM, Stack EC, Gjini E, Hu X, Redd R, Freeman GJ, Neuberg D, Hodi FS, Liu XS, Shipp MA, Rodig SJ. Topological analysis reveals a PD-L1 associated microenvironmental niche for Reed-Sternberg cells in Hodgkin lymphoma. Blood. 2017. 10.1182/blood-2017-03-770719 10.1182/blood-2017-03-770719 PMC5766840 28893733

[5] Koh YW, Han JH, Yoon DH, Suh C, Huh J (2017). PD-L1 expression correlates with VEGF and microvessel density in patients with uniformly treated classical Hodgkin lymphoma. Ann Hematol.

[6] Vrzalikova K, Ibrahim M, Vockerodt M, Perry T, Margielewska S, Lupino L, Nagy E, Soilleux E, Liebelt D, Hollows R, Last A, Reynolds G, Abdullah M, Curley H, Care M, Krappmann D, Tooze R, Allegood J, Spiegel S, Wei W, Woodman CBJ, Murray PG 2017 S1PR1 drives a feedforward signalling loop to regulate BATF3 and the transcriptional programme of Hodgkin lymphoma cells. Leukemia. 10.1038/leu.2017.275 10.1038/leu.2017.275 PMC5737877 28878352

[7] Cohen M, Vistarop AG, Huaman F, Narbaitz M, Metrebian F, De Matteo E, Preciado MV, Chabay PA (2017). Cytotoxic response against Epstein Barr virus coexists with diffuse large B-cell lymphoma tolerogenic microenvironment: clinical features and survival impact. Sci Rep.

[8] Adams CM, Mitra R, Gong JZ, Eischen CM (2017). Non-Hodgkin and Hodgkin lymphomas select for overexpression of BCLW. Clin Cancer Res.

[9] Jiang L, Si T, Yu M, Zeng X, Morse HC, Lu Y, Ouyang G, Zhou JX (2017). The tumor suppressive role of inhibin βA in diffuse large B-cell lymphoma. Leuk Lymphoma.

[10] Karube K, Enjuanes A, Dlouhy I, Jares P, Martin-Garcia D, Nadeu F, Ordóñez GR, Rovira J, Clot G, Royo C, Navarro A, Gonzalez-Farre B, Vaghefi A, Castellano G, Rubio-Perez C, Tamborero D, Briones J, Salar A, Sancho JM, Mercadal S, Gonzalez-Barca E, Escoda L, Miyoshi H, Ohshima K, Miyawaki K, Kato K, Akashi K, Mozos A, Colomo L, Alcoceba M, Valera A, Carrió A, Costa D, Lopez-Bigas N, Schmitz R, Staudt LM, Salaverria I, López-Guillermo A, Campo E 2017 Integrating genomic alterations in diffuse large B-cell lymphoma identifies new relevant pathways and potential therapeutic targets. Leukemia. 10.1038/leu.2017.251 10.1038/leu.2017.251 PMC5843901 28804123

[11] Bouska A, Bi C, Lone W, Zhang W, Kedwaii A, Heavican T, Lachel CM, Yu J, Ferro R, Eldorghamy N, Greiner TC, Vose J, Weisenburger DD, Gascoyne RD, Rosenwald A, Ott G, Campo E, Rimsza LM, Jaffe ES, Braziel RM, Siebert R, Miles RR, Dave S, Reddy A, Delabie J, Staudt LM, Song JY, McKeithan TW, Fu K, Green M, Chan WC, Iqbal J (2017). Adult high-grade B-cell lymphoma with Burkitt lymphoma signature: genomic features and potential therapeutic targets. Blood.

[12] Wei S, Perera MLW, Sakhtemani R, Bhagwat AS (2017). A novel class of chemicals that react with abasic sites in DNA and specifically kill B cell cancers. PLoS One.

[13] Fujisawa M, Sakata-Yanagimoto M, Nishizawa S, Komori D, Gershon P, Kiryu M, Tanzima S, Fukumoto K, Enami T, Muratani M, Yoshida K, Ogawa S, Matsue K, Nakamura N, Takeuchi K, Izutsu K, Fujimoto K, Teshima T, Miyoshi H, Gaulard P, Ohshima K, Chiba S Activation of RHOA-VAV1 signaling in angioimmunoblastic T-cell lymphoma. Leukemia. 10.1038/leu.2017.273 10.1038/leu.2017.273 PMC5843900 28832024

[14] Tokuhira M, Saito S, Okuyama A, Suzuki K, Higashi M, Momose S, Shimizu T, Mori T, Anan-Nemoto T, Amano K, Okamoto S, Takeuchi T, Tamaru JI, Kizaki M (2017). Clinicopathologic investigation of methotrexate-induced lymphoproliferative disorders, with a focus on regression. Leuk Lymphoma.

[15] Alonso-Álvarez S, Magnano L, Alcoceba M, Andrade-Campos M, Espinosa-Lara N, Rodríguez G, Mercadal S, Carro I, Sancho JM, Moreno M, Salar A, García-Pallarols F, Arranz R, Cannata J, Terol MJ, Teruel AI, Rodríguez A, Jiménez-Ubieto A, González de Villambrosia S, Bello JL, López L, Monsalvo S, Novelli S, de Cabo E, Infante MS, Pardal E, García-Álvarez M, Delgado J, González M, Martín A, López-Guillermo A, Caballero MD (2017). Risk of, and survival following, histological transformation in follicular lymphoma in the rituximab era. A retrospective multicentre study by the Spanish GELTAMO group. Br J Haematol.

[16] Shimono J, Miyoshi H, Kamimura T, Eto T, Miyagishima T, Sasaki Y, Kurita D, Kawamoto K, Nagafuji K, Seto M, Teshima T, Ohshima K (2017). Clinicopathological features of primary splenic follicular lymphoma. Ann Hematol.

[17] Paul U, Richter J, Stuhlmann-Laiesz C, Kreuz M, Nagel I, Horn H, Staiger AM, Aukema SM, Hummel M, Ott G, Spang R, Rosenwald A, Feller AC, Cogliatti S, Stein H, Hansmann ML, Moller P, Szczepanowski M, Burkhardt B, Pfreundschuh M, Schmitz N, Loeffler M, Trümper L, Siebert R, Klapper W (2017). Advanced patient age at diagnosis of diffuse large B-cell lymphoma is associated with molecular characteristics including ABC-subtype and high expression of MYC. Leuk Lymphoma.

[18] Virmani P, Levin L, Myskowski PL, Flores E, Marchetti MA, Lucas AS, Pulitzer M, Horwitz S, Trippett T, Moskowitz A, Querfeld C (2017). Clinical outcome and prognosis of young patients with mycosis fungoides. Pediatr Dermatol.

[19] Suzuki Y, Kato S, Kohno K, Satou A, Eladl AE, Asano N, Kono M, Kato Y, Taniwaki M, Akiyama M, Nakamura S (2017). Clinicopathological analysis of 46 cases with CD4(+) and/or CD56(+) immature haematolymphoid malignancy: reappraisal of blastic plasmacytoid dendritic cell and related neoplasms. Histopathology.

[20] Kezlarian B, Alhyari M, Venkataraman G, Karner K, Inamdar KV, Menon MP 2017 GATA3 Immunohistochemical staining in Hodgkin lymphoma: diagnostic utility in differentiating classic Hodgkin lymphoma from nodular lymphocyte predominant Hodgkin lymphoma and other mimicking entities. Appl Immunohistochem Mol Morphol. 10.1097/PAI.0000000000000581, 1 10.1097/PAI.0000000000000581 28877074

[21] Untanu RV, Back J, Appel B, Pei Q, Chen L, Buxton A, Hodgson DC, Ehrlich PF, Constine LS, Schwartz CL, Hutchison RE (2017). Variant histology, IgD and CD30 expression in low-risk pediatric nodular lymphocyte predominant Hodgkin lymphoma: a report from the Children’s Oncology Group. Pediatr Blood Cancer.

[22] Iwamuro M, Takenaka R, Nakagawa M, Moritou Y, Saito S, Hori S, Inaba T, Kawai Y, Toyokawa T, Tanaka T, Yoshino T, Okada H (2017). Management of gastric mucosa-associated lymphoid tissue lymphoma in patients with extra copies of the MALT1 gene. World J Gastroenterol.

[23] Zheng M, Perry AM, Bierman P, Loberiza F Jr, Nasr MR, Szwajcer D, Del Bigio MR, Smith LM, Zhang W, Greiner TC 2017 Frequency of MYD88 and CD79B mutations, and MGMT methylation in primary central nervous system diffuse large B-cell lymphoma. Neuropathology. doi: 10.1111/neup.12405 10.1111/neup.12405 28856744

[24] Nielsen JS, Chang AR, Wick DA, Sedgwick CG, Zong Z, Mungall AJ, Martin SD, Kinloch NN, Ott-Langer S, Brumme ZL, Treon SP, Connors JM, Gascoyne RD, Webb JR, Berry BR, Morin RD, Macpherson N, Nelson BH (2017). Mapping the human T cell repertoire to recurrent driver mutations in MYD88 and EZH2 in lymphoma. Oncoimmunology.

[25] Li X, Xu-Monette ZY, Yi S, Dabaja BS, Manyam GC, Westin J, Fowler N, Miranda RN, Zhang M, Ferry JA, Medeiros LJ, Harris NL, Young KH (2017). Primary bone lymphoma exhibits a favorable prognosis and distinct gene expression signatures resembling diffuse large B-cell lymphoma derived from centrocytes in the germinal center. Am J Surg Pathol.

[26] Pham-Ledard A, Prochazkova-Carlotti M, Deveza M, Laforet MP, Beylot-Barry M, Vergier B, Parrens M, Feuillard J, Merlio JP, Gachard N (2017). Molecular analysis of immunoglobulin variable genes supports a germinal center experienced normal counterpart in primary cutaneous diffuse large B-cell lymphoma, leg-type. J Dermatol Sci.

[27] Fernandez-Pol S, De Stefano D, Kim J (2017). Immunohistochemistry reveals an increased proportion of MYC-positive cells in subcutaneous panniculitis-like T-cell lymphoma compared with lupus panniculitis. J Cutan Pathol.

[28] Leclaire Alirkilicarslan A, Dupuy A, Pujals A, Parrens M, Vergier B, Robson A, Delfau-Larue MH, Ingen-Housz-Oro S, Chosidow O, Haioun C, Beylot-Barry M, Merlio JP, Copie-Bergman C, Gaulard P, Ortonne N (2017). Expression of TFH markers and detection of RHOA p.G17V and IDH2 p.R172K/S mutations in cutaneous localizations of angioimmunoblastic T-cell lymphomas. Am J Surg Pathol.

[29] van den Brand M, Rijntjes J, Hebeda KM, Menting L, Bregitha CV, Stevens WB, van der Velden WJ, Tops BB, van Krieken JH, Groenen PJ (2017). Recurrent mutations in genes involved in nuclear factor-κB signalling in nodal marginal zone lymphoma-diagnostic and therapeutic implications. Histopathology.

[30] van den Brand M, Balagué O, van Cleef PH, Groenen PJ, Hebeda KM, de Jong D, van Krieken JH (2015). A subset of low-grade B cell lymphomas with a follicular growth pattern but without a BCL2 translocation shows features suggestive of nodal marginal zone lymphoma. J Hematop.

[31] van den Brand M, Mathijssen JJ, Garcia-Garcia M, Hebeda KM, Groenen PJ, Falini B, Serrano S, van Krieken JH (2015). Immunohistochemical differentiation between follicular lymphoma and nodal marginal zone lymphoma—combined performance of multiple markers. Haematologica.

[32] Zamò A, Pischimarov J, Schlesner M, Rosenstiel P, Bomben R, Horn H, Grieb T, Nedeva T, López C, Haake A, Richter J, Trümper L, Lawerenz C, Klapper W, Möller P, Hummel M, Lenze D, Szczepanowski M, Flossbach L, Schreder M, Gattei V, Ott G, Siebert R, Rosenwald A, Leich E 2017 Differences between BCL2-break positive and negative follicular lymphoma unraveled by whole-exome sequencing. Leukemia. 10.1038/leu.2017.270 10.1038/leu.2017.270 28824170

[33] Igawa T, Omote R, Sato H, Taniguchi K, Miyatani K, Yoshino T, Sato Y (2017). A possible new morphological variant of mantle cell lymphoma with plasma-cell type Castleman disease-like features. Pathol Res Pract.

[34] Velders GA, Kluin-Nelemans JC, De Boer CJ, Hermans J, Noordijk EM, Schuuring E, Kramer MH, Van Deijk WA, Rahder JB, Kluin PM, Van Krieken JH (1996). Mantle-cell lymphoma: a population-based clinical study. J Clin Oncol.

[35] Abrisqueta P, Scott DW, Slack GW, Steidl C, Mottok A, Gascoyne RD, Connors JM, Sehn LH, Savage KJ, Gerrie AS, Villa D (2017). Observation as the initial management strategy in patients with mantle cell lymphoma. Ann Oncol.

[36] Eskelund CW, Dahl C, Hansen JW, Westman M, Kolstad A, Pedersen LB, Montano-Almendras CP, Husby S, Freiburghaus C, Ek S, Pedersen A, Niemann C, Räty R, Brown P, Geisler CH, Andersen MK, Guldberg P, Jerkeman M, Grønbæk K (2017). TP53 mutations identify younger mantle cell lymphoma patients who do not benefit from intensive chemoimmunotherapy. Blood.

[37] Zlamalikova L, Moulis M, Ravcukova B, Liskova K, Malcikova J, Salek D, Jarkovsky J, Svitakova M, Hrabalkova R, Smarda J, Smardova J (2017). Complex analysis of the TP53 tumor suppressor in mantle cell and diffuse large B-cell lymphomas. Oncol Rep.

[38] Visco C, Wang J, Tisi MC, Deng L, D’Amore ESG, Tzankov A, Montes-Moreno S, Dybkær K, Bhagat G, Hsi ED, van Krieken JH, Ponzoni M, Ferreri AJM, Møller MB, Piris MA, Medeiros LJ, Xu-Monette ZY, Young KH (2017). Hepatitis C virus positive diffuse large B-cell lymphomas have distinct molecular features and lack BCL2 translocations. Br J Cancer.

[39] Wu CC, Takahashi E, Asano N, Miyata-Takata T, Takata K, Furukawa K, Elsayed AA, Hu LM, Satou A, Kohno K, Kosugi H, Ohashi K, Kinoshita T, Nakamura S, Kato S (2017). Primary cutaneous NK/T-cell lymphoma of nasal type: an age-related lymphoproliferative disease?. Hum Pathol.

[40] Wen KW, Gill RM 2017 Immature terminal deoxynucleotidyl transferase positive B cells are detected in a subset of adult and pediatric liver biopsies. Appl Immunohistochem Mol Morphol. doi: 10.1097/PAI.0000000000000596, 1 10.1097/PAI.0000000000000596 28968264

[41] Kiesewetter B, Simonitsch-Klupp I, Kornauth C, Dolak W, Lukas J, Mayerhoefer ME, Raderer M 2017 Immunohistochemical expression of cereblon and MUM1 as potential predictive markers of response to lenalidomide in extranodal marginal zone B-cell lymphoma of the mucosa-associated lymphoid tissue (MALT lymphoma). Hematol Oncol. 10.1002/hon.2472 10.1002/hon.2472 28833354

[42] Bielska M, Borowiec M, Jesionek-Kupnicka D, Braun M, Bojo M, Pastorczak A, Kalinka-Warzocha E, Prochorec-Sobieszek M, Robak T, Warzocha K, Młynarski W, Lech-Marańda E (2017). Polymorphism in IKZF1 gene affects clinical outcome in diffuse large B-cell lymphoma. Int J Hematol.

[43] Lee YS, Liu J, Fricano KA, Webb EM, Toolsie DR, Jones S, Rhoads JA, Vij R, Cashen AF, Abboud CN, Westervelt P, Bartlett NL, Dipersio JF, Kreisel FH, Lim KH 2017 Lack of a prognostic impact of the MyD88 L265P mutation for diffuse large B cell lymphoma patients undergoing autologous stem cell transplantation. Biol Blood Marrow Transplant 10.1016/j.bbmt.2017.08.022 28847710

[44] Leivonen SK, Taskinen M, Cervera A, Karjalainen-Lindsberg ML, Delabie J, Holte H, Lehtonen R, Hautaniemi S, Leppä S (2017). Alternative splicing discriminates molecular subtypes and has prognostic impact in diffuse large B-cell lymphoma. Blood Cancer J.

[45] Kato T, Miyoshi H, Kobayashi S, Yoshida N, Imaizumi Y, Seto M, Uchimaru K, Miyazaki Y, Ohshima K (2017). Clinicopathological analysis in PTCL-NOS with CADM1expression. Virchows Arch.

